# Immunotherapy-related pneumonitis and bacterial pneumonia after the successful treatment of metastatic malignant melanoma with pembrolizumab: A case report

**DOI:** 10.1097/MD.0000000000024018

**Published:** 2021-01-08

**Authors:** Qin Ma, Lei Yang, Feng Gu

**Affiliations:** Department of Respiratory Oncology, Gansu Province Cancer Hospital, Lanzhou, 730050, China.

**Keywords:** malignant melanoma, pembrolizumab, pulmonary metastasis

## Abstract

**Introduction:**

Pembrolizumab, a monoclonal antibody targeting programmed cell death-1 (PD-1), is approved as a therapy for unresectable or metastatic melanoma. Immunotherapy-associated pneumonitis is an uncommon event.

**Patient concerns:**

A 73-year-old man was admitted to our hospital with a history of melanoma on the left side of the face (resected in December 2012) and metastasis to the left lung upper lobe (resected in November 2016). Recurrence of metastasis to the bilateral lungs and left pleura was detected in April 2018. A complete response was achieved following treatment with pembrolizumab, with lower limb rashes the only adverse events occurring during therapy. The patient was readmitted in March 2019 with a productive cough, shortness of breath, and mild fever, and sputum culture identified *Escherichia coli*.

**Diagnosis:**

A diagnosis of pneumonia was made, and although cough and shortness of breath responded to ceftazidime and levofloxacin, but fever and poor appetite persisted. Computed tomography showed no improvement in the bilateral lower lobe lesions. Prednisone was initiated based on a clinical diagnosis of immunotherapy-related pneumonitis. The response to prednisone confirmed the diagnosis.

**Interventions:**

The patient first received ceftazidime and levofloxacin, but the symptoms persisted. Prednisone was initiated based on a clinical diagnosis of immunotherapy-related pneumonitis.

**Outcome:**

Complete resolution of the bilateral lung lesions occurred after 45 days of prednisone therapy.

**Conclusion:**

This case report highlights that both pneumonitis and bacterial pneumonia can occur as complications of anti-PD-1 immunotherapy.

## 1 Introduction

Cutaneous melanoma is an aggressive cancer derived from melanocytes in the skin. The melanoma incidence rate in the USA was around 20 per 100,000 in 2011 but is predicted to rise during the next decade.^[1]^ In China, an estimated 20,000 people are diagnosed with melanoma annually, and the incidence is increasing by 3% to 5% each year.^[2]^ The management of melanoma involves extended excision of the primary lesion together with sentinel lymph node biopsy, regional lymph node dissection, interferon therapy, and/or palliative radiotherapy (for bone or brain metastasis), depending on the clinical stage.^[2]^ Nevertheless, melanoma is associated with a poor prognosis, and the 5-year overall survival rate is less than 25% in patients with metastatic (stage IV) disease.^[3,4]^

Recent years have seen major advances in immunotherapies for melanoma.^[5]^ Among these are monoclonal antibodies targeting programmed cell death-1 (PD-1) and cytotoxic T lymphocyte-associated antigen-4 (CTLA-4), both of which are receptors on T cells that inhibit T cell function and thus suppress cell-mediated killing of tumor cells.^[5]^ Pembrolizumab is a monoclonal antibody against PD-1 that has been approved by the U.S. Food and Drug Administration for the treatment of unresectable or metastatic melanoma.^[6]^ Several clinical trials^[7–11]^ and real-world studies^[12–14]^ have demonstrated that pembrolizumab is an effective and well-tolerated treatment for advanced melanoma.

This report describes the case of a 73-year-old man with pulmonary metastasis of cutaneous melanoma who responded well to pembrolizumab, but the patient subsequently developed symptoms attributed to both immune-related pneumonitis and bacterial pneumonia. This case report highlights that although pneumonitis and pneumonia are uncommon complications related to pembrolizumab therapy, both can develop concomitantly in patients with melanoma treated with anti-PD-1 immunotherapy.

## 2 Case report

This case report was approved by the Ethics Committee of Gansu Province Cancer Hospital. The patient provided written informed consent for the publication of this case report. A 73-year-old man was admitted to our hospital on December 27, 2016. The patient had undergone resection of a skin tumor on the left side of the face 4 years ago (December 2012), and postoperative pathology subsequently diagnosed the tumor as malignant melanoma of Clark level IV. No cancer cells were found at the resected margins, and the patient was treated with interferon and interleukin for more than 1 year after surgery. One month before admission to our hospital (November 2016), an isolated metastatic lesion about 1.5 cm in diameter was identified in the upper lobe of the left lung, and thoracoscopic resection of the left upper lobe was performed. Postoperative pathology confirmed a malignant tumor in the upper lobe of the left lung that was consistent with pulmonary metastasis of melanoma.

**Figure 1:**
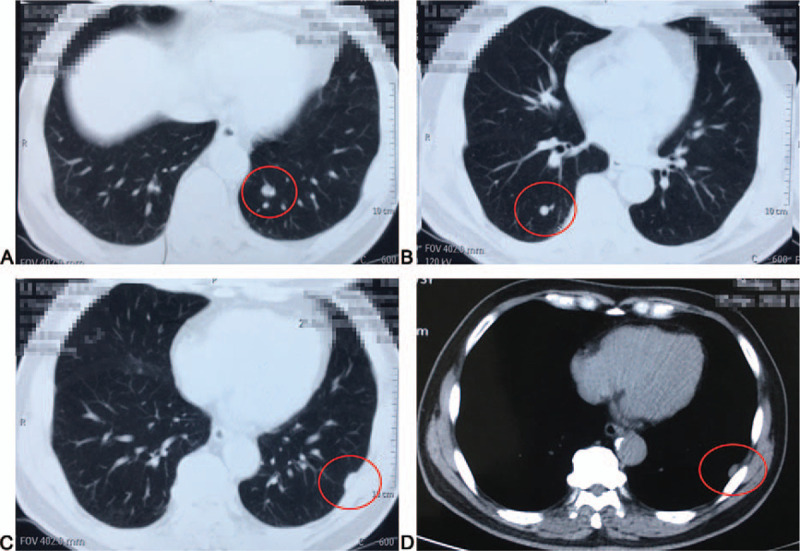
(A–D) Pulmonary computed tomography (CT) suggested bilateral lung and left pleural (red circle) metastases after a dacarbazine/interleukin-2 regimen for 4 cycles.

Routine blood investigations, liver and kidney function tests, electrocardiography, and other examinations conducted in our hospital on the day after admission (December 28, 2016) showed no abnormalities, and the patient was given a dacarbazine/interleukin-2 regimen for 4 cycles. Subsequent pulmonary computed tomography (CT) in April 2018 suggested bilateral lung and left pleural metastases (Fig. 1A–D). A paraffin section of the original metastatic lesion in the upper lobe of the left lung was sent on June 6, 2018, to determine the tumor mutation burden (TMB) using an Illumina TruSight Oncology 500 platform (Illumina, Inc., San Diego, CA). All exons of 288 genes, introns, promoters, or fusion breakpoints of 38 genes and some exons of 728 genes (including point mutation, small fragment insertion–deletion, copy number variation, and known fusion genes) are examined for TMB. The TMB was 23.0 Muts/Mb. The abundance of the BRAF p.G469R mutation abundance was 4%, with microsatellite stability. On June 20, 2018, CT examination showed slight enlargement of the original metastatic foci and metastasis to the left chest wall, and the chest wall mass was palpable on physical examination.

Pembrolizumab (200 mg every 3 weeks) was initiated. A partial response was observed after 4 cycles of treatment, and a complete response was achieved after 10 cycles. The only adverse event during the period of therapy was the appearance of a red rash scattered bilaterally on the lower limbs, but no treatment was required for this rash. No abnormalities were found in routine blood tests, liver, and kidney function tests, or the electrocardiogram.

**Figure 2:**
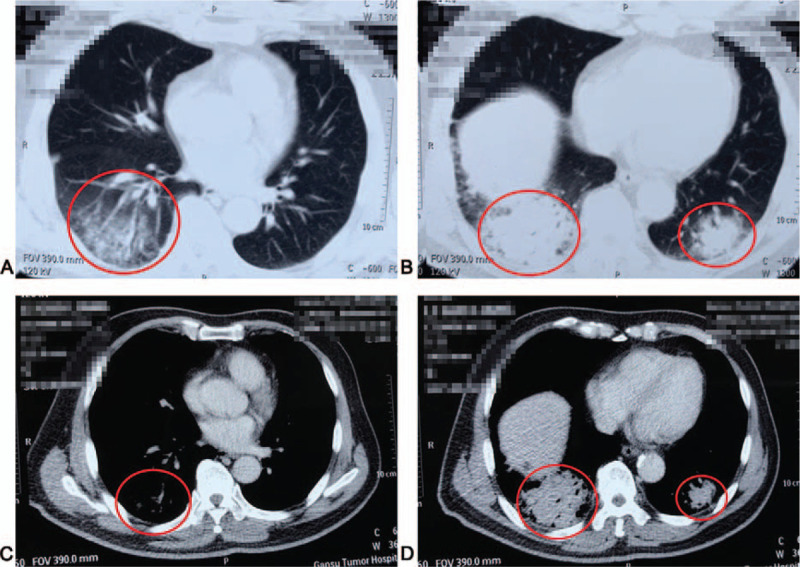
(A–D) CT demonstrated flocculent, cotton-like, high-density shadows (red circle) in the lower aspects of both lungs.

In late February 2019, the patient developed a mild cough with the production of a small amount of white mucus. His symptoms progressed to shortness of breath and mild fever (37.8°C), and he was readmitted on March 2, 2019. On admission, routine blood tests and liver and kidney function tests were normal. Sputum smear microscopy indicated the presence of Gram-negative bacteria, and sputum culture revealed the presence of *Escherichia coli*, without fungi. CT findings on March 5, 2019 (Fig. 2A–D) demonstrated flocculent, cotton-like, high-density shadows in the lower aspects of both lungs, consistent with a diagnosis of pneumonia.

**Figure 3:**
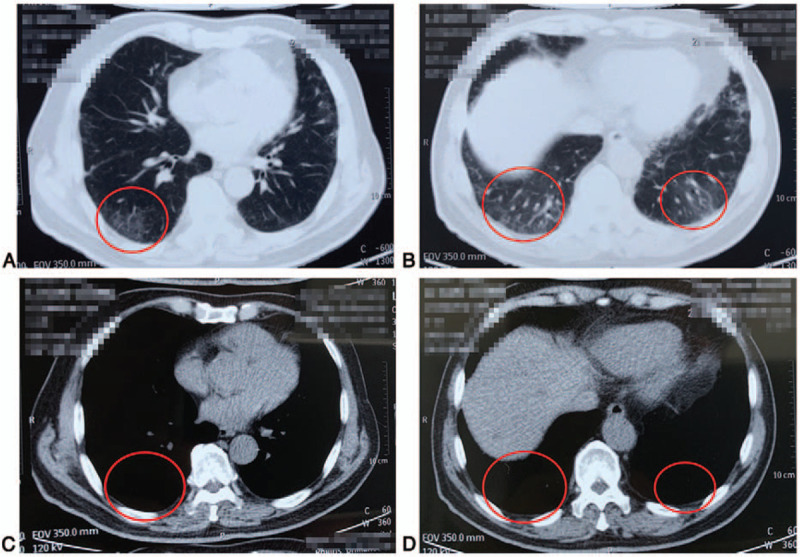
(A–D) CT examination after prednisone therapy indicated complete resolution (red circles) of the bilateral lung lesions.

Based on the antimicrobial susceptibility results, the patient was treated with ceftazidime (2 g i.v. b.i.d.) and levofloxacin (0.4 g i.v. o.d.). In addition, physical cooling and ambroxol hydrochloride were used as symptomatic treatments for cough and sputum production. Although the cough and shortness of breath were reduced after 3 days of treatment, the low-grade fever and poor appetite persisted. CT on March 18, 2019, showed no improvement of the lesions in the lower lobes of both lungs, as well as partial consolidation. Since no cause of pneumonitis apart from immunotherapy could be identified and since the clinical presentation was consistent with immunotherapy-related pneumonitis, levofloxacin was discontinued, and prednisone was added (20 mg p.o. b.i.d.). The patient's fever, shortness of breath, and lack of appetite began to resolve rapidly, and ceftazidime was also discontinued. The symptoms completely disappeared after 5 days of treatment with prednisone, and CT examination on April 15, 2019, revealed substantial resolution of the lesions in the lower lobes of the bilateral lungs. Prednisone therapy was continued for a total of 45 days, with drug withdrawal achieved by 5-mg dosage reductions every 3 days. CT examination on May 22, 2019, indicated complete resolution of the bilateral lung lesions (Fig. 3A–D).

Therefore, considering the poor response to antibiotics but the good response to prednisone, the diagnosis of immunotherapy-related pneumonitis was made since no other cause of pneumonitis could be identified in this patient, that is, he was taking no drug (except immunotherapy) known to be associated with pneumonitis, he had no repeated exposure to molds and bacteria, he had no direct exposure to birds, and he did not receive radiation therapy.

## 3 Discussion

Immune checkpoint inhibitors have become useful treatments for a variety of hematologic and solid malignancies.^[15]^ PD-1 and CTLA-4 are receptors found on T cells, and activation of these receptors by their ligands leads to T cell down-regulation.^[5]^ Tumor cells express ligands for PD-1 (PD-L1) and CTLA-4 (B7), and the interaction of these tumor ligands with the respective T cell receptors results in T cell suppression, which allows tumor cells to evade immune cell-mediated killing.^[5]^ Numerous previous clinical studies have reported that pembrolizumab, a monoclonal IgG4-kappa monoclonal antibody with a high affinity for PD-1, is an effective immunotherapy for advanced melanoma.^[7–14]^ Consistent with these previous investigations, the present case exhibited a good response to pembrolizumab, with complete resolution of the metastatic pulmonary lesions after 10 cycles of treatment. It has proven difficult to identify biomarkers that predict the effect of immunotherapy due to the complexities of the immune signaling pathways and interactions between immune cells, cytokines, and immune adjuvants in the tumor microenvironment. TMB is one factor considered to be related to the response to anti-PD-1 therapy.^[16–18]^ In the present case, TMB expression was high (≥10 Muts/Mb), and this may have contributed to the excellent response to treatment with pembrolizumab.

Immune checkpoint inhibitors are associated with adverse reactions that differ from those of conventional chemotherapy and targeted molecular therapy. Since tumors have similar antigens to those of normal cells, the activation of T cells can result in normal tissue cells also being attacked. The production of autoantibodies and inflammatory factors and activation of the complement system can cause immune-related inflammation in normal tissues.^[19]^ Immune-related adverse events can occur in almost all tissues and organs and are most common in the skin, intestines, endocrine organs, liver, and lungs, with only rare involvement of the heart, nervous system, and eyes.^[20]^ The most common adverse events associated with pembrolizumab are diarrhea, nausea, pruritus, maculopapular rash, arthralgia, fatigue, and hypothyroidism, while hyperthyroidism, hepatitis, hypophysitis, adrenal insufficiency, neuropathy, Guillain-Barré syndrome, myasthenia gravis, and nephritis have been reported less frequently.^[8,9,11,13,20,21]^ In the present case, the only adverse event detected during therapy with pembrolizumab was a rash on the lower limbs, and this did not require treatment.

Pneumonitis is an uncommon but potentially fatal adverse effect of anti-PD-1 therapy.^[22]^ Pneumonitis related to anti-PD-1 therapy occurs with an incidence of about 5%, but the incidence of grades 3 to 4 pneumonitis is only around 1% to 2%.^[8,9,11,13,22,23]^ The main clinical manifestations of immunotherapy-related pneumonitis are cough, chest pain, and dyspnea, although a few patients can be asymptomatic and present only with pulmonary imaging changes such as ground-glass opacities, mesh-like shadows, and non-specific interstitial pneumonia-like changes. Pneumonia secondary to infection has also been described in patients given anti-PD-1 therapy.^[24]^ In the present case, pulmonary imaging revealed cotton-like, high-density shadows located at the sites of melanoma metastasis, and sputum culture identified the presence of a pathogen (*E coli*). Based on the clinical features and response to therapy (initially with antimicrobial drugs and then with steroid), it was concluded that the patient, in this case, had a co-existing pulmonary bacterial infection and immune-related pneumonitis. The patient's symptoms and abnormal imaging findings were completely resolved after treatment with ceftazidime, levofloxacin, and prednisone, and his general clinical condition remained good during follow-up. Previous case reports have concluded that pneumonitis with concomitant bacterial pneumonia is associated with a poorer prognosis than pneumonitis without co-existing bacterial pneumonia.^[25]^ This emphasizes the importance of correctly diagnosing and treating co-existent pneumonitis and pneumonia in patients receiving immune checkpoint inhibitors such as pembrolizumab.

The take-away message of this case report is that pneumonia can be complicated by immunotherapy-related pneumonitis in cancer patients treated with immunotherapy. Immunotherapy-related pneumonia is not very common, with a frequency of about 5%,^[22]^ while the incidence of grades 3 to 4 pneumonitis is only 1% to 2%.^[21]^ Therefore, the diagnosis of pneumonia is more probable when a patient presents with pulmonary symptoms. In this case, sputum culture results were positive. Antibiotics were partly effective in reducing the symptoms, but not the radiological images. Immunotherapy-related pneumonitis was then suspected, and prednisone was given, with complete resolution of the pulmonary symptoms, confirming the diagnosis of pneumonitis.

## Author contributions

**Data curation:** Lei Yang.

**Investigation:** Lei Yang.

**Methodology:** Qin Ma.

**Resources:** Feng Gu.

**Writing – original draft:** Qin Ma, Feng Gu.
